# Failure Analysis of Retrieved Osteosynthesis Implants

**DOI:** 10.3390/ma13051201

**Published:** 2020-03-07

**Authors:** Mihai Nica, Bogdan Cretu, Dragos Ene, Iulian Antoniac, Daniela Gheorghita, Razvan Ene

**Affiliations:** 1 University of Medicine and Pharmacy Carol Davila Bucharest, 050474 Bucharest, Romania; mikx99n@gmail.com (M.N.); razvan77ene@yahoo.com (R.E.); 2 Orthopedics and Traumatology Department, Bucharest Emergency University Hospital, 050098 Bucharest, Romania; jfrbogdan@yahoo.com; 3 Surgery Department, Emergency Clinical Hospital of Bucharest, 050098 Bucharest, Romania; 4 Politehnica University of Bucharest, 060042 Bucharest, Romania; antoniac.iulian@gmail.com

**Keywords:** osteosynthesis, failure, analysis, implant, retrieval

## Abstract

Failure of osteosynthesis implants is an intricate matter with challenging management that calls for efficient investigation and prevention. Using implant retrieval analysis combined with standard radiological examination, we evaluated the main causes for osteosynthesis implant breakdown and the relations among them for a series of cases. Twenty-one patients diagnosed with implant failure were assessed for this work. For metallurgical analysis, microscopy techniques such as scanning electron microscopy (SEM), stereomicroscopy, and optical microscopy were employed. The results showed that material structural deficiencies (nine patients) and faulty surgical techniques (eight patients) were the main causes for failure. An important number of patients presented with material structural deficiencies superimposed on an imperfect osteosynthesis technique (six patients). Consequently, the importance of failure retrieval analysis should not be overlooked, and in combination with other investigational techniques, must provide information for both implant manufacturing and design improvement, as well as osteosynthesis technique optimization.

## 1. Introduction

Failure analysis is of great importance not only for orthopedics [1,2,3,4,5], but also for other various medical areas such as general surgery [6,7,8,9], gynecology [10,11,12], cranioplasty [13,14], ophthalmology [15], and dentistry [16,17], as the investigation of retrieved implants offers insight into implant failure mechanisms and how to prevent such cases. Physiologic forces are transmitted to intact human bone under normal conditions without exceeding its ultimate strength. When non-physiologic forces are applied by trauma or normal bone strength is affected by various pathological entities, fractures ensue. There are different forces acting on the human skeleton, with natural physiologic loading comprising a combination of these. A fracture line is characterized by abnormal stress and strain which must be neutralized by stabilizing devices (external or internal) in order to provide favorable conditions for bone healing [18,19].

Internal fracture fixation, or osteosynthesis, uses metallic implants that have to meet specific requirements for orthopedic surgical use. These requirements refer to design, size, and material properties like strength, stiffness, resistance to fatigue, biocompatibility, and corrosion resistance [20,21,22]. Last but not least, pre-operative management plays an essential role and needs to be taken into account for favorable outcomes, as Table 1 presents. Implants used for osteosynthesis are manufactured using iron-based alloys such as surgical grade stainless-steel 316L, titanium-based alloys, cobalt-chromium-based composites, or shape-memory alloys [23,24,25]. Of these, stainless-steel 316L is the most commonly used, due to its cost-effectiveness and good integration of corrosion resistance and mechanical properties.

**Figure 1:**
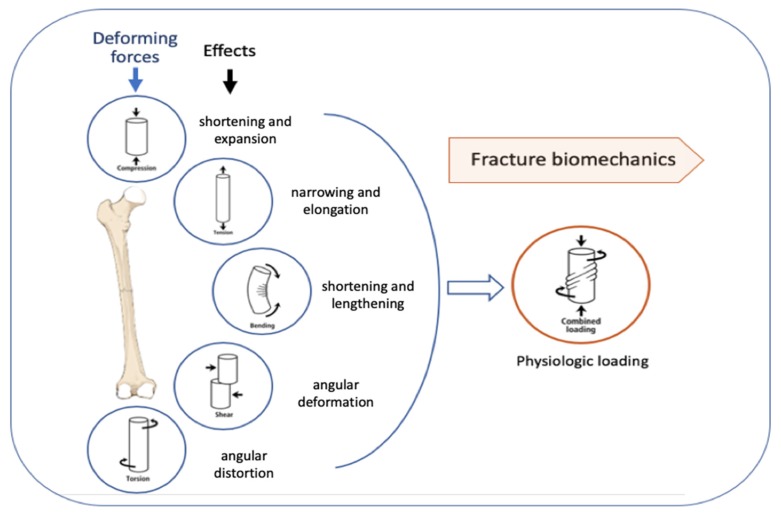
Deforming forces acting on long bones.

Successful clinical results are also dependent on adherence to sound osteosynthesis principles which achieve load sharing between the implant and bone during the healing period. The main implant failure mechanisms are mechanical or biomechanical [26]. A numerical analysis can also provide useful information regarding the loading forces that act on the bone and on the couple formed by a bone-osteosynthesis implant [27,28]. The tolerable mechanical stress level of an implant may be exceeded either through cyclic loading (fatigue) or under a single critical load (static or dynamic), combined with the corrosive effects of the internal biological environment. Especially for fatigue management, a good load-sharing level between bone and implant is crucial, which is contingent on good reduction of fracture and proper structural support, provided by adequately-reduced bone fragments [29,30]. Figure 1 presents the biomechanics of fractures and how forces act on the bone.

Therefore, both surgical technique and the level of bone comminution—which impacts structural support—become very important elements for reaching an equilibrium of forces needed for stable and reliable fixation until healing ensues. If this balance of forces is not established and maintained, areas of local stress concentration may appear on the implant surface and failure can occur. Biologic failure manifests as implant loosening caused by bone resorption at the contact interface with the implant, due to an inappropriate stabilizing device, excessive physiologic loading during injudicious rehabilitation, or prolonged bone healing (biologic factors play a key role) [31,32,33].

This paper presents the failure analysis of a series of stainless-steel osteosynthesis implants used for primary fixation and revised in the Orthopedics Department of Bucharest Emergency University Hospital Bucharest.

## 2. Materials and Methods

In one year, approximately eight hundred cases required osteosynthesis in the Orthopedics Department of the Bucharest Emergency University Hospital. The reported number of cases with osteosynthesis implant failure was just under 1%, which is consistent with reported rates in the literature. Twenty-one cases of osteosynthesis implant failure diagnosed and managed in our department during a three-year period were assessed for this work. All cases had the primary fixation surgery also done in our department, with a time-to-failure period ranging from four weeks to seven months. The majority of patients were male (19 cases), with an age span between 18 and 78 years. Table 2 presents the centralized data of patients with osteosynthesis implant failure.

In terms of location, 10 were femoral implants, 5 tibia, and 6 humerus implants. The femoral implants used were 3 Gamma nails, one interlocking nail, and 6 dynamic hip screws. Cases with diagnosed infection and pathological fractures were not included in the study because of the confounding role they can have on research [34,35,36]. The osteosynthesis performed was by closed reduction and intramedullary nailing in 6 cases (3 femoral and 3 tibia fractures), one of the femoral fracture sites, and one tibia fracture requiring open reduction, followed by nailing. The humerus lesions were managed by open reduction and internal fixation with reconstruction or dynamic compression plates and screws (Auxein Medical, Haryana, India) in 3 cases or closed reduction and intramedullary nailing for the other 3. All implants (Auxein Medical, Haryana, India) used were made of austenitic stainless-steel type 316L, implanted using standard instrument sets and surgical techniques.

**Figure 2:**
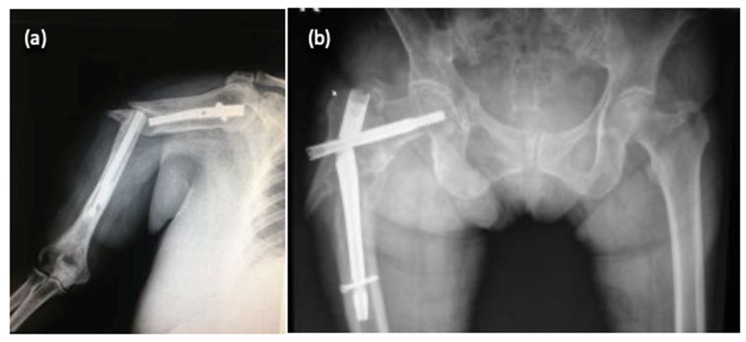
Radiological aspects of fractures with deterioration of osteosynthesis material: humerus fracture (**a**) and femoral fracture (**b**).

Diagnosis of implant failure was confirmed either by standard radiological examination during regular follow-up visits, or on admission for patients with inciting events and new symptoms generated by implant deterioration. Some radiographic images showing the aspect of deteriorated osteosynthesis implants are presented in Figure 2.

Images were assessed focusing on quality of reduction, implant type adequacy, size, and position. Retrieval of broken implants was performed with special care not to cause more damage, especially to the fracture surface.

Implant failure investigation was conducted by means of macroscopic evaluation, optical microscopy, and scanning electron microscopy in concordance with recommended standards of retrieval analysis of failed internal fixation devices.

## 3. Results

Regarding external factors for osteosynthesis breakdown, we identified nine cases with critical loading during single traumatic events and five patients with injudicious rehabilitation and excessive loading. Subsequent radiological evaluation revealed that in eight cases (38%), fracture reduction was flawed with malrotation, which can add to the dynamic loading of implants or an interfragmentary gap of more than 2 mm. Only two cases (9.5%) had an unsuitably-sized implant, but always with an external factor added to the failure process of the fixation device.

### 3.1. Metallographic Analysis

**Figure 3:**
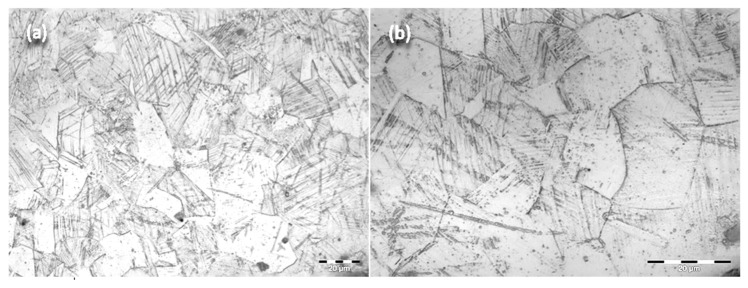
Optical microscopy images of experimental samples obtained from retrieved intramedullary nails made of stainless-steel type 316L: 500 × (**a**), 1000 × (**b**).

Optical microscopy images obtained with an Olympus BX51 (Olympus Soft Imaging Solutions, Münster, Germany,) highlighted the metallographic structure of the sample, as seen in Figure 3. The presence of recrystallization slabs inside the crystalline grains is noted, and inhomogeneous grains of variable size are observed. The obtained images do not give reason to claim that the failure of the sample was due to technical errors, as no impurities and no gaps are present in the material.

### 3.2. Stereomicroscopy

**Figure 4:**
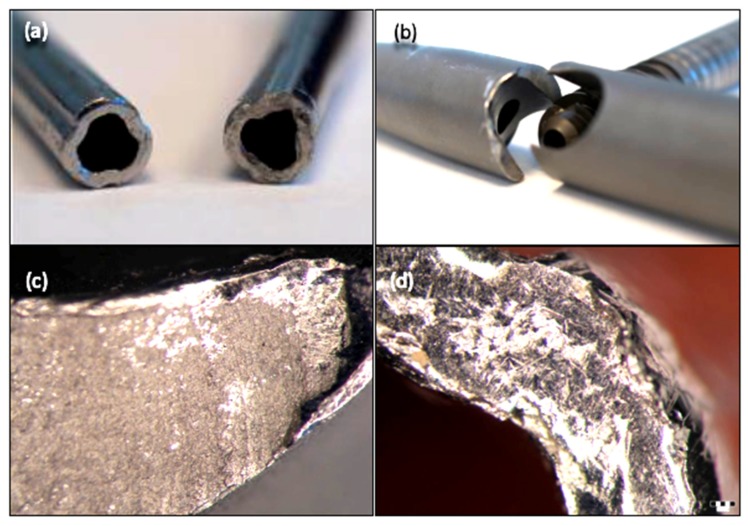
The macroscopic aspect (**a**,**b**) and stereomicroscopy images (**c**,**d**) of the fractured zone of some retrieved intramedullary nails.

Following stereomicroscopy analysis performed on the fracture section, the image of the fracture zone can be observed (Figure 4c,d). Analysis was made using a Olympus SZX7 stereomicroscope (Olympus Soft Imaging Solutions, Münster, Germany). There are different areas with distinct aspects throughout the entire surface of the fracture. Some areas present an intergranular fracture with secondary cracks and a fibrous matte appearance, while other areas (glossy appearance) have a bright crystalline appearance and indicate the site of fracture initiation. Therefore, the fracture has a mixed character specific to a fatigue fracture with initiation, propagation, and sudden final fracture.

### 3.3. Scanning Electron Microscopy

**Figure 5:**
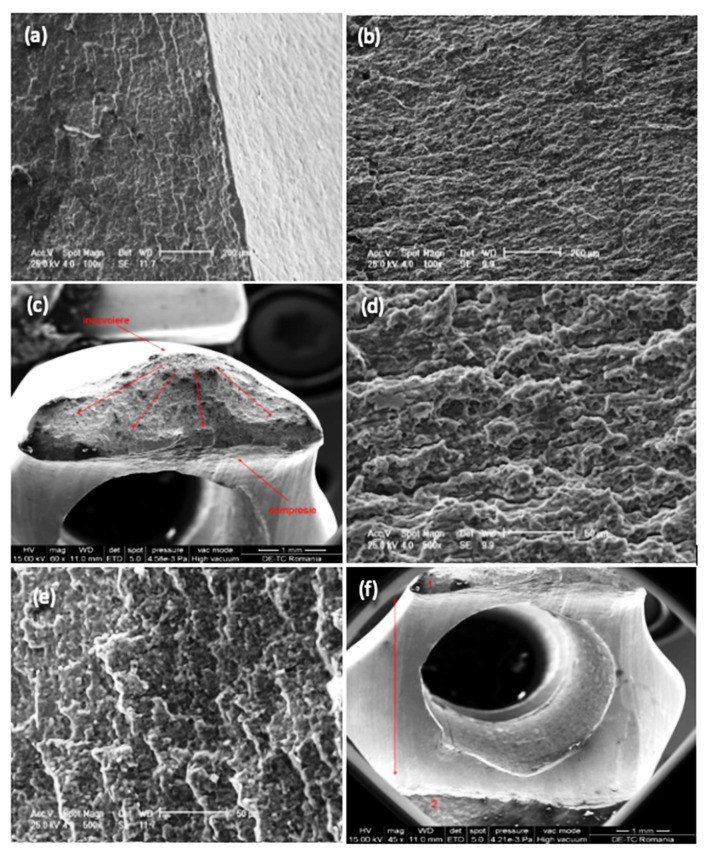
Scanning electron micrographs showing fracture surface morphology on failure region for retrieved intramedullary nails: 100 × (**a**,**b**), 60 × (**c**), 500 × (**d**,**e**), 45 × (**f**).

SEM (Philips model ESEM XL 30 TMP-FEI Company, Eindhoven, Netherlands) was used to characterize the surface morphology of the fracture site. Analysis of the images obtained with the scanning electron microscope (SEM) reinforces the results obtained from optical microscopy analysis. The fracture had a mixed character, and the propagation waves of the crack can be clearly observed (Figure 5).

After implant failure analysis, the results showed that nine (42.85%) devices had structural abnormalities with either material inclusions inside the superficial layer, or surface defects with evidence of corrosion—all of which accounted for initiation points for material fracture (Table 2). The cause for surface defects could not be clearly attributed to either damage caused by a careless surgical implantation techniques or to engineering process errors. Material composition evaluation revealed elemental compositions with values compatible with the standard specifications for surgical grade stainless-steel 316L.

## 4. Discussion

Successful bone fracture treatment by means of osteosynthesis is dependent on a myriad of factors, such as correct indication and implant choice, surgical technique, appropriate rehabilitation, biological factors, and implant characteristics. Nevertheless, fixation devices are not intended for long-term load bearing in vivo, so the fracture healing process must restore the physiological equilibrium of forces and reclaim the stress-bearing role of the intact bony structure.

When mechanical failure of fixation devices occurs, it can be categorized as fatigue failure (under cyclic loading), plastic, or brittle. Fatigue and brittle breakdown are usually associated with flaws in the material or design of the implant. Fatigue combined with corrosion represents a fearsome and frequent failure cause, especially for stainless-steel internal fixation devices.

**Figure 6:**
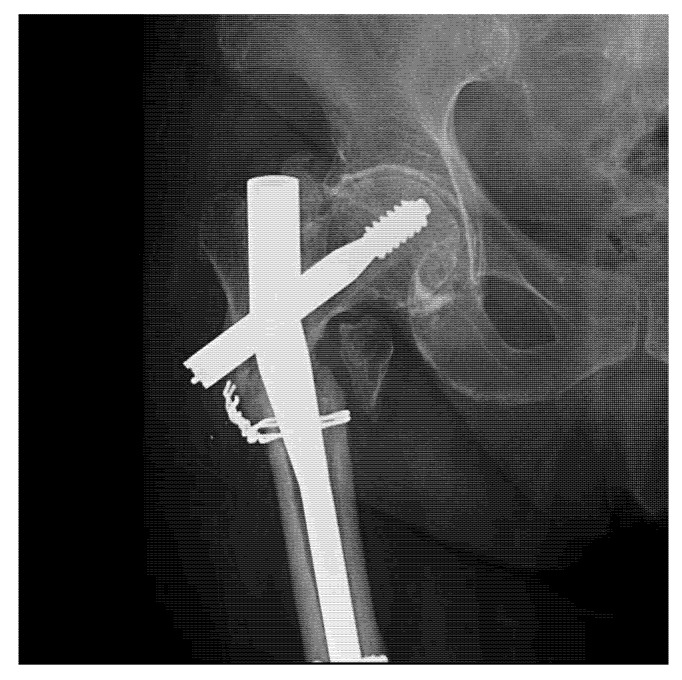
Radiography of a clinical case with failure of a Gamma Nail—72-year-old female patient.

For intramedullary nails, it is well-recognized that the failure process is usually initiated in the locking holes, proximally or distally (case presented in Figure 6). Sliding screw hip plates are prone to significant wear and corrosion due to moving components [37,38]. For plates, the breakdown starting point is commonly situated around the holes where the cross-sectional area is reduced and where interaction with the screw heads generates wear and corrosion fatigue under localized stress concentration [39].

Implant surface integrity is very important because it conventionally becomes the initiation point for implant fracture. Damage to the surface may be attributed to careless surgical implantation techniques, dynamic loading conditions (fatigue), wear at component junctions, or faulty manufacturing. Every type of surface defect, e.g., pitting, fretting, fatigue cracking, or crevicing, is in danger for corrosion attack in vivo, which increases even more the risk for failure [40].

Unevenly distributed mechanical load on the implant may be considered a crucial element of the failure process and commonly stems from improper device choice or faulty implantation technique with inadequate reduction of the bone fragments and insufficient structural support.

Therefore, we consider that the two main factors that generate the failure process of osteosynthesis implants are structural abnormalities, with focus on implant surface integrity (initiation point) and inappropriate surgical treatment algorithm (from implant choice to surgical implantation technique). The second may be easily identified in clinical practice, but failed implant analysis for identification of structural abnormalities does not represent a standard procedure. This may mean that many intrinsic structural flaws of the material or ones inflicted during implantation may be overlooked, and therefore failure attributed to other causes. We also consider that these two causes coexist for a large percent of implant failures.

As seen in our series, a considerable number of cases exhibit imperfect reduction, which calls for improvement of surgical technique, but we believe that failure should not be attributed only to this factor, with the metallurgical analysis results supporting our view. Of the eight failure cases with recognized fracture reduction shortcomings, six patients also showed structural abnormalities upon retrieved implant analysis.

Although a rare event, an implant breakdown before adequate bone healing requires a challenging management with repeated surgeries, added risks for complications, higher costs, and increased social and psychological burdens for the patient. Implant failure can manifest as various types of material fracture patterns, depending on the underlying mechanism. This is why legislation and protocols governing the manufacture, trade and use of osteosynthesis implants must be well- established and implemented, ensuring the best quality and reliability and that the relation between material structural abnormalities and surgical treatment flaws are better analyzed. Furthermore, retrieval analysis of failed devices used for internal fracture fixation, which provides useful data for manufacturing process optimization and surgical protocols development, must become a more prevalent tool for failure research and anticipation [41,42,43,44].

The results of our research show that the main two causes for implant failure are inadequate surgical technique and intrinsic material deficiencies, coupled with corrosion. All these can induce and promote failure initiation points and result in material breakdown.

## 5. Conclusions

Intramedullary nail failure represent a problematic matter with an even more demanding management. Albeit a rare occurrence, it brings significant human and financial costs, calling for continuous research and development of all factors involved in failure prevention.

Along with surgical implantation techniques and device manufacturing improvements, retrieval analysis of failed implants is an important tool that can provide data for development and implementation of better quality standards. This is supported by the results of our research, which show that structural material flaws account for a high percentage of the observed causes of failure. In order to identify the breaking mechanisms of osteosynthesis devices, explant analyses are needed. Macroscopic and microscopic investigations, including stereomicroscopy and scanning electron microscopy, are techniques that can help us identify the causes that lead to failure of implants.

## Figures and Tables

**Table 1:** Pre-operative planning.

Radiographic Images (Multiple Views)	Implant Length	Implant Diameter
Bone morphology	Contralateral bone x-rays (magnified)	IM canal isthmus (the narrowest portion of the canal)
Shape of the intramedullary (IM) canal	Traction radiographs	1.0 to 1.5 mm greater than anticipated IM nail diameter
Fracture pattern and comminution	Distance between palpable bony landmarks	

**Table 2:** Statistics data on patients with osteosynthesis implant failure.

No.	Patient Age	Fracture Location (Bone Type)	Type of Implant	Failure Causes				
				External Factors (Traumatic Event)	Surgical Causes		Implant Defects	
					Inadequate Implant Size	Deficient Fracture Reduction	Materials Defects	Surface Defects
***1***	**36**	**Tibia**	**IMN**	**(+)**				
***2***	65	Femur	IMN			**(+)**		**(+)**
***3***	72	Femur	GN	**(+)**				
***4***	26	Humerus	PS				**(+)**	
***5***	44	Tibia	IMN	**(+)**	**(+)**	**(+)**		**(+)**
***6***	31	Tibia	PS	**(+)**		**(+)**	**(+)**	
***7***	71	Femur	GN	**(+)**				
***8***	67	Femur	DHS			**(+)**		**(+)**
***9***	39	Humerus	IMN	**(+)**	**(+)**			
***10***	78	Femur	DHS	**(+)**				
***11***	46	Tibia	IMN	**(+)**		**(+)**	**(+)**	
***12***	42	Humerus	PS	**(+)**				**(+)**
***13***	69	Femur	DHS			**(+)**		
***14***	18	Humerus	IMN	**(+)**				
***15***	68	Femur	DHS					
***16***	32	Humerus	PS	**(+)**				**(+)**
***17***	74	Femur	GN	**(+)**				
***18***	59	Femur	DHS	**(+)**				
***19***	46	Humerus	IMN			**(+)**		**(+)**
***20***	55	Tibia	IMN	**(+)**				
***21***	69	Femur	DHS			**(+)**		
***IMN=Intramedullary Nail; GN=Gamma Nail; DHS=Dynamic Hip Screw; PS=Plate-Screw System***								
